# A Plague among the Violets

**Published:** 1882-01

**Authors:** 


### A PLAGUE AMONG THE VIOLETS.

Another interesting problem for microscopists to solve is the cause of the disease which has broken out among the violets, an account of which was lately given by a leading florist.

When the disease commenced its ravages, some three years ago, violet growing was so far in the hands of a single producer that he had won the titular dignity of the violet king among New York florists. His vast plantation was wrecked in one summer, and he was financially prostrated by the operations of an invisible enemy. The season had been rather dry, and the blight was attributed in this special instance to the substitution of well for brook water in irrigating the plants. Experience soon furnished an emphatic negative to this theory, and showed that the disease was a true blight, like the potato rot, the vine disease, the pear tree blight, and similar destructive agencies that infest the vegetable kingdom. In the violet the disease makes its appearance while the plants are in blossom. The first symptom is the development of nearly circular spots on the petals of the flower, which resemble the spots caused by the concentration of the beams of the sun upon the surfaces of the leaves of plants by the refractive agency of raindrops after a summer shower, the globular and lenticular shape of the drop rendering it equivalent to a minute burning glass, concentrating the rays of the summer sun upon the surface beneath, and completely destroying the delicate vessels thus exposed to intense heat. After this symptom appears, the destruction of the plant is a question of a few hours only ; the leaves become limp and wilted, the stem withers from the root, and the delicate organism is soon transformed, from the minutest rootlet to the tip of the leaf, into a dry and lifeless effigy. The origin and natural history of the violet blight have not yet been investigated.—*Scientific American.*

